# Embryonic remnants of intercentra and cervical ribs in turtles

**DOI:** 10.1242/bio.20135439

**Published:** 2013-09-04

**Authors:** Ingmar Werneburg, Wolfgang Maier, Walter G. Joyce

**Affiliations:** 1 Geowissenschaften, Universität Tübingen, Hölderlinstrasse 12, 72076 Tübingen, Germany; 2 Paläontologisches Institut und Museum, Universität Zürich, Karl-Schmid-Strasse 4, 8006 Zürich, Switzerland; 3 Zoologisches Institut, Spezielle Zoologie, Universität Tübingen, Auf der Morgenstelle 28, 72076 Tübingen, Germany; 4 Department für Geowissenschaften, Universität Fribourg, Chemin du Musée 6, 1700 Fribourg, Switzerland

**Keywords:** Testudines, Development, Blastema, Ribs, Vertebrae, Pleurodira

## Abstract

A broad sample of extant turtles possesses a series of paired bones in the neck that are situated between the cervical vertebrae. These paired bones were originally proposed to be cervical rib remnants, but have more recently been interpreted as vestiges of intercentra. Here, we document, for the first time, the neck development of a pleurodire turtle, *Emydura subglobosa*, and identify blastematous structures, which partially recapitulate the ribs and intercentra of the plesiomorphic tetrapod condition. We identify blastematous “bridges” between intercentra and the corresponding ribs, which we homologize with the vestiges visible in extant turtles and with the remnant parapophyseal articulation processes of the intercentra of some stem taxa. Only the unpaired, median part of the intercentrum of the atlas is retained in adult turtles, but intercentra are recapitulated along the entire vertebral column during development; they are embedded in the cervical myosepta and serve as attachment sites for neck musculature. We also identify two rib rudiments in the occipital region, which may indicate that at least two vertebrae are integrated into the cranium of turtles in particular, and of amniotes in general.

## Introduction

**Fig. 1.**
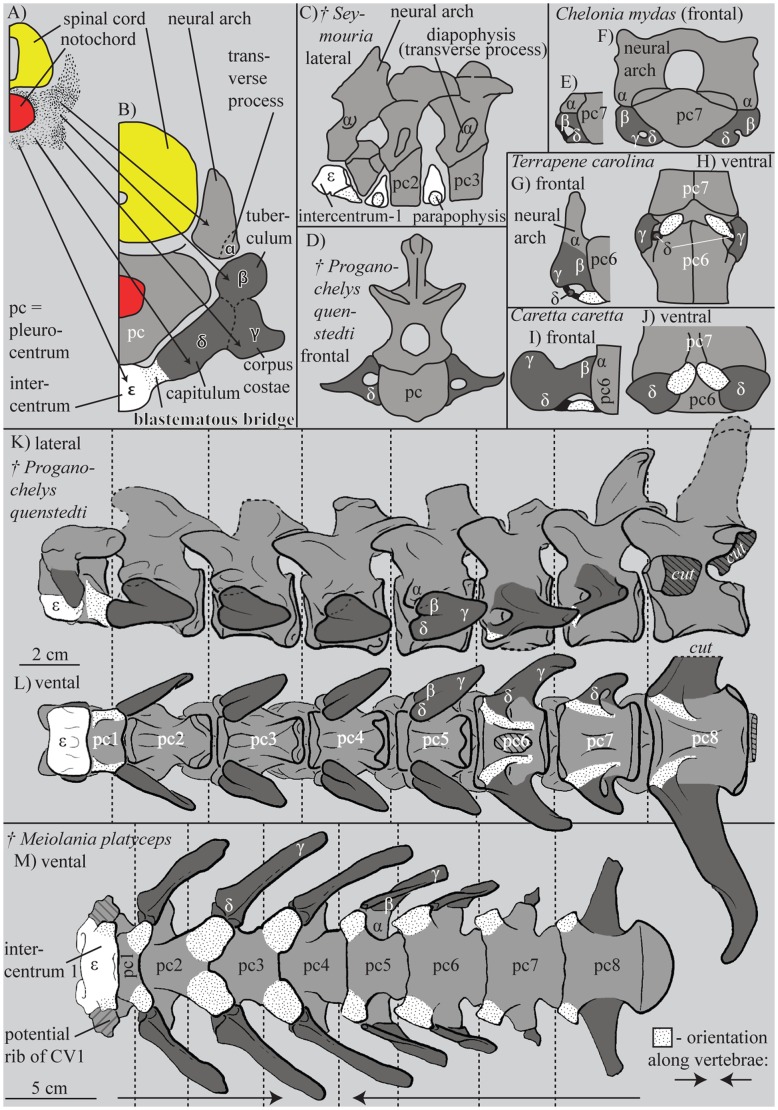
Comparison of neck vertebrae. (A,B) Scheme of the development of the vertebral region in a turtle with the terminology defined herein. (C) The first three neck vertebrae of *Seymouria sp.* (plesiomorphic amniote condition) (after Starck, 1979). Panels A,B,D–I were modified after Williams (Williams, 1959). (E–J) ‘Rib’ rudiments in different adult cryptodiran turtles. (K–M) Stem turtle necks (after Gaffney, 1985; Gaffney, 1990). The parapophyses of the intercentra are dotted. Whether a rib is actually present at the atlas of *Meiolania platyceps* is not certain (M, Gaffney, 1985) and is illustrated as “potential rib” herein.

The fossil record of turtles reveals that unambiguous representatives of the turtle stem lineage, such the Late Triassic *Proganochelys quenstedti*, had well-developed cervical ribs (Fig. 1D,K,L) (Gaffney, 1985; Gaffney, 1990; Li et al., 2008), whereas the ancestral crown turtle had extremely reduced cervical ribs (Joyce, 2007; Anquetin, 2012; Sterli and de la Fuente, 2013). A broad sample of extant turtles (listed by Williams, 1959), nevertheless, possesses a series of paired osseous structures that are associated with the intercentral joints of the neck (Fig. 1E–J; stippled) and that have puzzled embryologists and palaeontologists alike. The most extensive study of these bones was undertaken by Williams, who homologized these structures with the proximal parts of the ancestral, bicipital rib (Williams, 1959). Williams was furthermore able to demonstrate that these structures occur in a much broader sample of extant turtles than had previously been anticipated (i.e. all turtles to the exception of Trionychia) and that most osteological specimens in museums lack them, because they are easily lost during preparation (Williams, 1959). Gaffney more recently homologized these paired ossifications with the enlarged parapophyses of the basal fossil turtle *Meiolania platyceps* (Gaffney, 1985). Given that *M. platyceps* is otherwise known to have well-developed cervical ribs, Gaffney interpreted the paired bones as remnants of the intercentra instead (Gaffney, 1985). The presence of paired intercentral structures in turtles is unusual, however, as intercentra are only known to occur as unpaired structures in adult tetrapods (Fischer, 2010).

The two primary groups of extant turtles, Pleurodira and Cryptodira, differ in the way they retract their necks. Pleurodires withdraw their necks along the horizontal plane, whereas cryptodires fold their necks along a vertical plane (Joyce, 2007). It is to be expected that the reduction or loss of cervical ribs could be functionally connected with these retraction mechanisms. Further, considering that these two types of retraction most probably evolved independently, it is to be expected that the specific modes of reduction were in some ways different as well. However, all previous studies focused on cryptodires only, but this may have simply been a problem of sampling. The purpose of this study is therefore to describe, for the first time, aspects of the neck development of a pleurodire.

## Materials and Methods

**Fig. 2.**
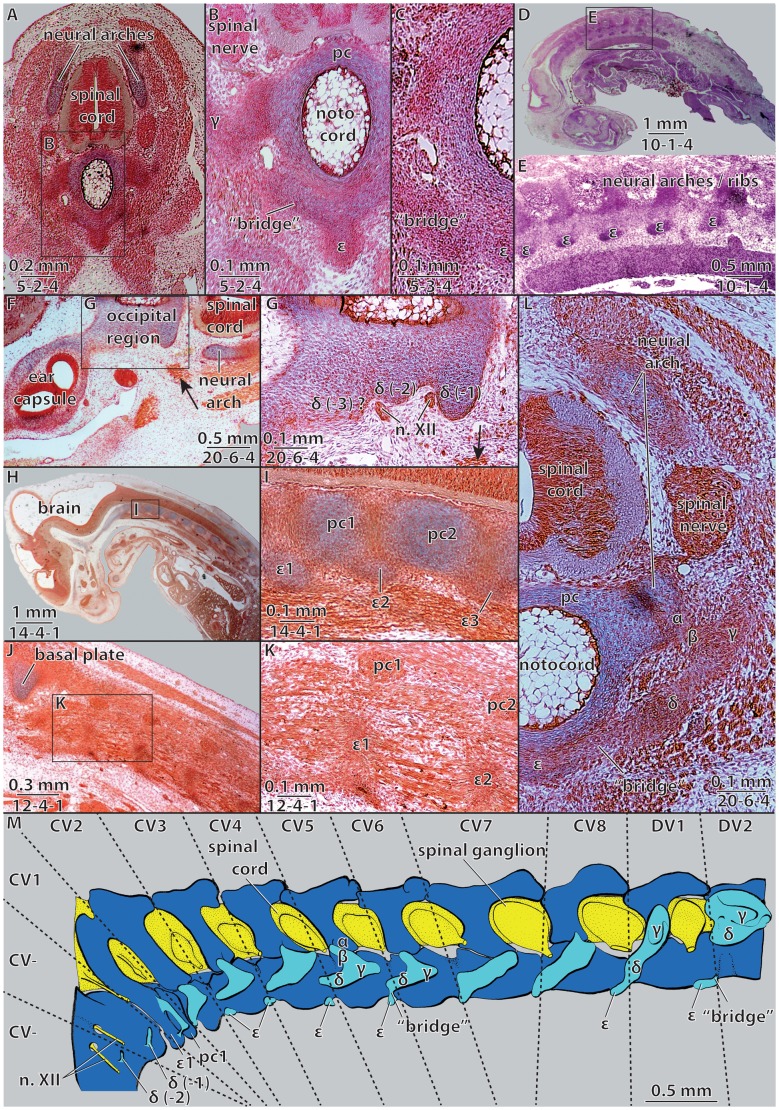
Embryonic neck anatomy of *Emydura subglobosa*. Embryonic vertebrae and ribs in specimens of (A,B) CL = 6.5 mm carapace length, (C,D) CRL = 10.5 mm, and (F–M) CL = 7.5 mm. (M) Redrawn 3 d reconstruction: dark blue  =  cartilaginous neck vertebrae, light blue  =  blastematous condensations. Numbers under scale bars indicate section numbers. (A–C) Cross sections through a cervical vertebra with partly developed embryonic ribs; (D,E) sagittal section through the whole body with (E) a focus on the neural arches/ribs; (F,G) cross section through the left ear capsule, the occipital and the anterior cervical region with (G) a focus on the occipital skull region. (H–K) Sagittal section through the anterior part of the body with (I,J) a focus on the first cervical vertebrae, H/I  =  mid sagittal and J/K  =  more lateral; (L) cross section through cervical vertebra six, compare to panel M. CV, cervical vertebra; n. XII, branches of nervus hypoglossus. For further abbreviations see Fig. 1B. Arrows in panels F,G indicate unisegmental neck muscle attaching to the posterior most occipital rib (δ-1).

We studied the embryonic neck of the pleurodire *Emydura subglobosa* during the blastematous stage of cervical development (Fig. 2) and compared our findings to those of Williams (Williams, 1959). Meaningful specimens are difficult to obtain because axial development takes place during a narrow temporal window (compare to SES-stages of Werneburg et al., 2009). The histological sections used in this study were stained with Azan after Haidenhain and are housed in the Zoological Collection of W.M. (Fachbereich Biologie at Universität Tübingen: specimen 1: carapace length (CL) = 6.5 mm; specimen 2: crown rump length (CRL) = 10.5 mm; specimen 3: CL = 7.5 mm). A three-dimensional reconstruction of the neck of *E. subglobosa* (specimen 3) was built using a modified plate reconstruction technique using polystyrene foam boards of 2 mm thickness; the drawing is based on this model (Fig. 2M). All blastematous condensations were delineated from the sections by W.M. without any knowledge of turtle neck anatomy. The results are therefore not the circular confirmation of *a priori* expectations. The interpretation is further supported by the work of Howes and Swinnerton (Howes and Swinnerton, 1901). They found comparable embryonic cell condensations in the vertebral column of the tuatara, a species which processes intercentra in the adult.

## Results and Discussion

The embryonic ribs of *Emydura subglobosa* consist of three parts (Fig. 1A): tuberculum (β), corpus costae (γ), and capitulum (δ). Each rib is dorsally articulated with the transverse process of the neural arch (α) and ventrally connected with the intercentrum (ε) via a blastematous bridge, which is more continuous with ε than with δ (Fig. 1B, Fig. 2L). The transverse process (α) is part of the neural arch. The most differentiated ribs of the neck are clearly bicipital and situated in the middle third of the cervical column (Fig. 2L,M). Whereas the cartilages of the neural arch and pleurocentrum are clearly distinct, all blastematous structures characteristically grade into one another. Well-pronounced concentrations in cell density nevertheless exist. Based on topological criteria we interpret these as vestigial skeletal elements (*sensu* Howes and Swinnerton, 1901).

Our reconstruction shows that there may be at least two, perhaps three vertebrae, which fuse to the occipital region of the skull to contribute to the parachordal region. Posteroventral to the two foramina nervi hypoglossi (Gaffney, 1972), two small, ventrolateral, cartilaginous processes are visible, which we homologize with capituli (δ) using topological criteria; the last projection still appears to be loosely connected with a short, unisegmental neck muscle (Fig. 2F,G; arrows).

The anlage of the first cervical vertebra shows a similar process, which, however, is broader when compared to the slender capituli (δ) associated with the occipital cranium. The capituli of the first cervical ribs are furthermore associated with the intercentrum of the first vertebra. The pleurocentrum of the first vertebrae is situated posterior to the intercentrum. Dorsally, the neural arches of the first and the second vertebrae appear to be fused. Additionally, neural arch 1 is fused to intercentrum 1 and not to the pleurocentrum, as in the other vertebrae.

The ribs of the second and eighth vertebrae show the typical differentiation into capitulum, tuberculum, and corpus costae. Ventromedially, they are connected to the intercentrum via a blastematous bridge. The capituli (δ) seem to be the last elements to be formed during development (Fig. 2A–C). The ribs of vertebrae three to five are also well differentiated, but they do not directly contact the related intercentra. The rib of vertebra six is very similar to the second rib, but the connection with the intercentrum (ε) is rather slender when compared to the other neck segments. Rib seven is ventrally connected to the intercentrum, its attachment to the neural arch is loose, and the tuberculum (β) is absent.

The ribs of the first and second dorsal (trunk) vertebrae are more differentiated (Fig. 2M), particularly their corpora costae (γ). Whereas the first trunk rib retains contact with its intercentrum, the second and the following trunk ribs are completely detached from their intercentra. Compared to the cervical ribs, the trunk ribs are situated more dorsally and are associated with the developing carapace (Fig. 2D,E,M). Except for the intercentrum of the atlas, no intercentrum or rib elements were documented in post-hatching specimens of *E. subglobosa* (Werneburg et al., 2009; Werneburg, 2011).

In the plesiomorphic, rhachitomous tetrapod condition (Fig. 1C), an anterior intercentrum (synonym: hypocentrum) and an almost equally sized posterior pleurocentrum form the vertebral body (Fischer, 2010; Pierce et al., 2013). The intercentra of early reptiliomorphs are reduced to 40% or less of the length of the pleurocentra (Lee and Spencer, 1997) (Fig. 1C) and the intercentrum is completely reduced or only rudimentarily present in amniotes (Fischer, 2010). The ribs of tetrapods usually show two articulations: the capitulum (δ) articulates with the intercentrum (ε) whereas the tubercle (β) articulates with the diapophysis (transverse process) of the neural arch (α); when the intercentrum has disappeared completely, the capitulum may be in contact with the parapophysis (Fischer, 2010). In adult turtles, only the intercentrum of the atlas is unambiguously present and only the pleurocentra form the bodies (“centra”) of all other vertebrae (Gaffney, 1990).

Williams recognized up to three fragmentary ‘rib’ elements in some adult turtle specimens, which he homologized with blastematous rib parts he observed in cryptodiran embryos (cf. Fig. 1A,B) (Williams, 1959). According to Williams, the transverse process (our α) is either formed by a separate chondrification or emerges from the same chondrification as the neural arch, depending on the species (Williams, 1959). For the pleurodire studied herein, we can confirm a common cartilaginous origin of the transverse process with the neural arch, which indicates that the former does not represent a fused rib element. Williams also identified a middle part of the rib (our γ) and a ventral part (our δ), which can persist as separated ossicles into adulthood (Fig. 1G,H) (Williams, 1959). We identified two further blastematous structures in the embryo, the anlage of the intercentrum (ε) and the closely associated blastematous bridge. The ventral ‘rib’ part described by Williams most likely represents the paired remnant of the blastematous bridges based on topological criteria (Williams, 1959).

The blastematous rib anlagen of our pleurodire embryos show diverse anatomical differentiations along the neck. Williams described only one stage (Williams, 1959), which could either indicate that synchronous rib development occurs in cryptodires or that the author described a somewhat more advanced ontogenetic state in which the other blastematous parts were already reduced. In the former case, a functional correlation with the retraction mode may be the cause. The blastematous rib of the second cervical vertebra in the pleurodire we studied closely resembles the cryptodire embryonic rib shape documented by Williams with its β, γ, and δ parts (Williams, 1959). A major difference is found in the expansion of the ventral-most part of the rib anlage. Whereas it ends with part δ in cryptodires (Williams, 1959), it connects to the intercentrum (ε) in the pleurodire via a blastematous bridge. By positional criteria and its close continuity to the intercentrum (ε), we suggest homology of these “bridges” with the parapophyseal processes of the intercentrum and with the paired bones seen in many adult specimens of different taxa (Fig. 1C,G–J); however, paired bones have not yet been documented for adult specimens of *E. subglobosa* specimens per se (Werneburg, 2011).

We furthermore homologize the blastematous bridges with the articulation (parapophyseal) processes of the intercentrum of early tetrapods. Although the unpaired intercentra are reduced in the stem turtle *Meiolania platyceps*, the processes are still present in that taxon as expanded parapophyses that articulate with the capitulum (δ) of the rib (Fig. 1M). Gaffney homologized the parapophyses of *M. platyceps* with the paired bones seen in the necks of many extant turtles (Gaffney, 1985) and we agree with that assessment based on our embryological evidence.

The variable modes of fusion of the intercentral parapophyses with the pleurocentra of *M. platyceps* support our proposed homology: the intercentrum and its associated parapophyses are formed ventrally and ventrolaterally between the adjacent pleurocentra (Fig. 2), and, after reduction of the intercentra, the parapophyses later fuse to the anterior (*M. platyceps*: vertebrae 1–3) or to the posterior, developmentally related (*M. platyceps*: cervical vertebrae 5–8) pleurocentra, or they remain as separated structures in extant taxa (Fig. 1G–J). The original “articulation” of the rib to the intercentrum (Fischer, 2010) is only recapitulated in the first neck segment (atlas), which retains its unpaired intercentrum.

The parapophyses of the intercentra appear to be the most important remainders of the vertebral appendages (‘ribs’ sensu Williams, 1959) in extant turtles. The musculi longus colli consist of numerous muscle bundles and are attached to these parapophyseal remnants (Werneburg, 2011). We speculate that the “ossified bridge-parts” are retained in reduced form among many extant turtles because they mechanically serve as insertion points within the highly mobile neck of living turtles or as developmental “aggregation points” for muscle fibres during development (Fig. 2H–J). Because ribs are by definition skeletal structures of the myosepta, it is not surprising that the rib blastemata are laterally continued by the mesenchyme of their myosepta (cf. Fig. 2K).

It is apparent that fully formed cervical ribs, such as those developed in *Proganochelys quenstedti*, would be incompatible with any retraction mechanism of the neck; it therefore represents a plesiomorphic evolutionary state. Conversely, turtles reduced the cervical ribs to achieve any kind of neck retraction. Current phylogenies of fossil turtles (Joyce, 2007; Anquetin, 2012; Sterli and de la Fuente, 2013) agree that adult specimens of at least some basal crown turtles (e.g. representatives of Xinjiangchelyidae; W.G.J., personal observations) still possessed reduced cervical ribs. It is therefore almost certain that extant cryptodires and pleurodires reduced their cervical ribs independently. However, with the present state of knowledge and scarcity of embryonic data, it seems premature to speculate about minor details of convergence and on features of heterochrony.

The developmental contribution of vertebrae to the formation of the primary neurocranium of vertebrates is generally accepted, mainly due to the numbers of roots of the hypoglossal nerve (Fürbringer, 1897; Starck, 1979). The primary neurocranium in actinopterygian fishes has recently been shown to include three occipital segments (Britz and Johnson, 2010). To our knowledge, occipital ribs have only been documented within Tetrapoda for the penguin *Spheniscus demersus* (Crompton, 1953). The presence of at least two occipital vestigial rib-homologues in the pleurodiran turtle *E. subglobosa* therefore suggests that at least two neck vertebrae are fused to the skull in sauropsids.
